# Aging, adaptation and maladaptation

**DOI:** 10.3389/fragi.2023.1256844

**Published:** 2023-08-28

**Authors:** Thomas Lissek

**Affiliations:** Interdisciplinary Center for Neurosciences, Heidelberg University, Heidelberg, Germany

**Keywords:** aging, age, adaptation, maladaptation, plasticity, longevity, transcription

## Abstract

Aging is accompanied by a dysregulation of adaptive processes. On the one hand, physiological adaptation mechanisms such as learning and memory, immune system plasticity and exercise-dependent muscle remodeling are blunted. On the other hand, several maladaptive processes increase with age including cancer, pathological cardiovascular remodeling and metabolic dysregulation. With increasing age the quotient of beneficial adaptation (Ab) to harmful adaptation (Ah), Ab/Ah, decreases. The adaptation-maladaptation framework of aging entails that there are age-related pathological phenotypes that are the result of activation of physiological adaptation mechanisms (e.g., maladaptation as a result of misdirection of adaptive cascades and molecular damage incurred by adaptation processes) and their occurrence over time might, to some degree, be inevitable. Aging might hence result from the organism’s inability to solve the adaptation-maladaptation dilemma. The present work explores the concept of counteracting aging through adaptation and proposes that interventions such as exercise, environmental enrichment and dietary restriction work in counteracting aging because they increase the ratio Ab/Ah by both raising Ab (e.g., by inducing metaplasticity in cells, meaning they raise the adaptability of cells to future stimuli) and decreasing Ah (e.g., through desensitizing certain potentially harmful adaptive mechanisms). Molecules whose aging-related expression changes can explain aspects of dysfunctional adaptation such as CREB and certain immediate early genes are examined and it is delineated how a better understanding of the dynamical organization of adaptation cascades could elucidate the seemingly complex role of adaptation in driving aging as well as protecting against it.

## Introduction

One of the major symptoms of aging in animals is a decreased body-wide ability for adaptation, which expresses itself as impaired neural plasticity (Burke and Barnes, 2006), decreased adaptive immune function (Weng, 2006) and blunted muscle anabolism (Breen and Phillips, 2011) among others. As adaptation is a fundamental property of organism function, its reduction leads to profound physiological impairments and in older humans is expressed, among other things, in reduced cognitive flexibility (Hedden and Gabrieli, 2004), decreased mobility (Rantakokko et al., 2013) and increased susceptibility to infectious diseases (Gavazzi and Krause, 2002). At the same time, during aging many maladaptive processes increase including dysfunctional remodeling of metabolism in diabetes (Sinclair et al., 2015), autoimmunity (Goronzy and Weyand, 2012), changes in the cardiovascular system such as atherosclerosis (Head et al., 2017)) as well as oncogenesis (White et al., 2014).

The present work explores how dysregulated adaptation is a central feature in animal aging and provides a framework to integrate various findings on the biology of aging at different levels of biological organization from the molecular to the behavioral. The adaptation-maladaptation framework of aging focuses on underlying functional patterns in living systems instead of concrete molecular or cellular entities and can integrate different biological scales of organization.

## Aging and a decrease in the ratio of beneficial to harmful adaptation

One of the central points of the present framework is that a fundamental phenomenon in aging is the decrease in the ratio of beneficial adaptation (Ab) to harmful adaptation (Ah) - Ab/Ah. As the organism ages, this ratio decreases consistently at various levels, from cells to tissues to the whole organism. Living systems and their subsystems become progressively less able to adapt to challenges from the environment (e.g., reduced adaptive immunity and cognitive memory) but instead display increasingly dysfunctional adaptation (e.g., systemic immune dysregulation and metabolic remodeling).

At the cellular level, a core aspect of mammalian aging is a decrease in plasticity which leads to a decrease in Ab. In humans, memory performance is impaired with increasing age (Luo and Craik, 2008) and in animal models loss of memory function is closely correlated to a decrease in neural plasticity in the brain, such as in synaptic LTP (Burke and Barnes, 2006). Exercise-dependent muscle anabolism is blunted in older humans (Breen and Phillips, 2011; Endo et al., 2020) which renders countermeasures against sarcopenia and its related risks difficult. Immune function is compromised with age, a phenomenon termed immunosenescence (Aiello et al., 2019), and adaptive cellular immunity is reduced with age (Weng, 2006), leading to increased rates of infection (Gavazzi and Krause, 2002). Furthermore, regeneration after injury is compromised in older individuals such as in reduced wound healing (Gosain and DiPietro, 2004). A decrease in beneficial cellular plasticity is hence a central feature of aging across multiple organ systems.

Conversely, many adaptive processes are misdirected in age-related disorders leading to an increase in Ah, with for instance adaptive transcription being involved in maladaptations such as cancer, metabolic dysregulation and autoimmunity (Lissek, 2022b). A central feature of cancer is the remarkable adaptability of cancer cells (Hanahan and Weinberg, 2011). They can evade treatment efforts and the immune system and modify their metabolism to survive under changing environmental conditions such as hypoxia or altered tissue physiology during metastasis. Another example for maladaptation is the increase in multi-organ inflammation mediated by the immune system with aging, sometimes referred to as “inflammaging” (Franceschi et al., 2018). In this process, a normally adaptive mechanism (i.e., recognition of pathogens and subsequent orchestration of an immune response) becomes misdirected and harms bodily tissues. Similarly, autoimmunity has been shown to increase with age (Goronzy and Weyand, 2012). Another dysfunctional adaptation process is the complex of insulin resistance and type 2 diabetes (DeFronzo et al., 2015) in which chronic stimuli such as certain types of nutrition and lack of exercise lead to harmful bodily adaptations. Thus, while beneficial adaptation decreases with age, certain maladaptive processes increase.

## Adaptive transcription components as potential causative agents for dysregulated adaptation in aging

It was previously outlined how adaptive transcription involving molecules such as cAMP-response element binding protein (CREB) is central to mediating the anti-aging effects brought on by physiological stimulation and several interventions such as blood transfer from young to old animals (Lissek, 2022a).

An interesting observation is that these molecular adaptation mediators are oftentimes reduced with age, potentially explaining the reduction in Ab. Previous research has shown that CREB levels decrease in the rodent brain with age (Brightwell et al., 2004) and increasing CREB levels in aged rodents leads to an increase in memory performance (Yu et al., 2017). Similarly, the activity-induced protein DNA-methyltransferase 3 A2 (Dnmt3a2) is reduced in the brains of aged mice and its overexpression rescues memory (Oliveira et al., 2012). Upstream regulators of adaptive transcription such as N-methyl D-aspartate receptor subtype 2B (NR2B)-containing NMDA receptors in the brain are also reduced during aging which correlates with memory deficits (Clayton et al., 2002). Interestingly, NR2B downregulation in certain brain areas seems to be a normal part of development (Liu et al., 2004), possibly due to a need for stabilization of neural circuits after heightened plasticity, highlighting how reductions in Ab might have physiological roles. In addition, a study has shown that aging is accompanied by a dysregulation in chromatin plasticity, such as for instance altered histone H4 lysine 12 (H4K12) acetylation in the brain which leads to blunted induction of a transcriptional program for memory formation and is associated with aging-related cognitive decline (Peleg et al., 2010).

On the side of Ah, several adaptive transcription mediators have been shown to be involved in maladaptive processes. Oncogenesis is promoted by CREB (Jean et al., 1998; Abramovitch et al., 2004; Aggarwal et al., 2008) and CREB regulated transcription coactivator 2 (CRTC2) (Rodon et al., 2019). Insulin resistance is driven by CREB (Qi et al., 2009) and CRTC2 (Hogan et al., 2015). Myocyte enhancer factor 2 (MEF2) is involved in cancer progression (Schwieger et al., 2009; Zhang et al., 2015; Xiao et al., 2021) and pathological cardiac remodeling (Kim et al., 2008). Fos proto-oncogene (Fos) has been implicated in oncogenesis (Saez et al., 1995; Racca et al., 2019), atherosclerosis (Miao et al., 2022) and inflammaging in the liver and kidneys (Yu et al., 2023). These examples demonstrate that adaptive transcription components which mediate cellular plasticity under physiological conditions can also be involved in driving aging-associated maladaptation (see previous work for a more detailed discussion (Lissek, 2022b)).

Another factor is that adaptation processes generally seem to work via potentially harmful mechanisms. Adaptive transcription induction in neurons for instance, which is required for memory formation, causes DNA double strand breaks (DSBs) (Madabhushi et al., 2015) and DSBs have been linked to the aging process (White and Vijg, 2016). Neural plasticity seems to involve formation of reactive oxygen species (ROS) (Massaad and Klann, 2011) and ROS have been connected to aging-associated physiological decline (Balaban et al., 2005). Aging-related damage could hence partly be a byproduct of continued activation of physiological adaptation mechanisms.

## Methods to increase Ab/Ah

One way to increase Ab is to engage adaptive programs via subjecting the body to physiological challenges including exercise, environmental enrichment and dietary restriction. Previous work has explored how these interventions counteract aging through the activation of adaptive gene programs (Lissek, 2022a). Environmental enrichment seems to induce metaplasticity in neurons (Buschler and Manahan-Vaughan, 2012), thus increasing Ab and the level of plasticity to future neuronal stimulation. Similarly, muscle cells express metaplasticity in which training for muscle hypertrophy at a young age facilitates muscle anabolism in older age (Gundersen, 2016).

A related way to raise Ab is to directly overexpress adaptive transcription components. In many cases, the overexpression of adaptive transcription molecules leads to enhancement of physiological function in processes such as learning and memory and exercise. Aging-related memory deficits can be reduced by overexpression of adaptation regulators such as CREB (Yu et al., 2017) and Dnmt3a2 (Oliveira et al., 2012) or upstream regulators such as calcium/calmodulin dependent protein kinase IV (CaMKIV) (Fukushima et al., 2008) and NR2B (Cao et al., 2007) among others. A third way is via pharmacological compounds. Molecules that act via CREB signaling and histone deacetylase (HDAC) inhibition have been shown to improve memory (Vecsey et al., 2007; Fass et al., 2013). Additionally, several artificial interventions have been shown to activate adaptive gene programs such as CREB activation through blood transfer from young to old animals (Villeda et al., 2014) and SRF activation after cerebrospinal fluid (CSF) transfer from young to old animals (Iram et al., 2022).

The decrease in Ah might be difficult to achieve without blunting Ab. One way could be by desensitizing adaptive processes through engaging them acutely. Many adaptation processes are desensitized after initial activation (e.g., neuronal depolarization leads to induction of immediate early genes and a subsequent reduction in their inducibility (Rienecker et al., 2022)) which could lead to a diminished availability of these genes for maladaptive processes and could explain the reduction in Ah in processes such as exercise and dietary restriction.

## Predictions and open questions

The adaptation-maladaptation framework of aging entails several points and predictions. The first is that the same entities (e.g., certain genes and molecules) and processes (e.g., defined cellular plasticity mechanisms) can be implicated in protection against aging, as well as in driving aging-related pathologies (e.g., CREB signaling in neuroprotection (Sakamoto et al., 2011) and cancer (Sakamoto and Frank, 2009)). As such, it should not be surprising when researchers find that certain processes and molecules that have been shown previously to be implicated in driving aging might also counteract aging depending on the circumstances such as for instance temporal activation patterns of molecular signaling cascades. The same molecules can have very different effects depending on the underlying dynamics such as transient vs sustained activation of signaling cascades (see reviewed in (Purvis and Lahav, 2013)). Some interventions might cause benefits and harm at the same time. For instance, the induction of some adaptive and protective mechanisms is coupled to molecular damage (e.g., environmental enrichment is neuroprotective (Young et al., 1999) but also leads to increases in DNA double strand breaks (Suberbielle et al., 2013)). Another point is that aging is conceptualized as a dysregulation of active processes and not merely the passive accumulation of damage. Thus, aging could potentially be reprogrammed and slowed down if one could decode adaptation goal states at various levels (cells, cell networks, organs, *etc.*) and understand how to direct, restrict and boost adaptation processes which has been previously discussed (Lissek, 2022b).

An overarching question is why the shift in Ab/Ah with increasing age happens in the first place. Developmental mechanisms could be connected to the decreases in Ab (e.g., decreasing plasticity to allow stable organism function in maturity as previously outlined (de Magalhaes and Sandberg, 2005)) as well as the increases in Ah (e.g., maladaptation through hyperfunction (Blagosklonny, 2013)). Perhaps the organism also implements a program to blunt adaptive cascades to reduce Ah and cannot do so without decreasing Ab. The increase in Ah could perhaps also partly be a result of aberrant activation of adaptation mechanisms through changes in overall transcription activity which has been shown to be dysregulated in aging (Debes et al., 2023).

Perhaps negative effects from the passage of time in biological organisms, and hence aging, might be inevitable. On the one hand, if the organism does not engage its adaptation mechanisms, certain types of molecular damage are reduced (e.g., activity-dependent DNA strand breaks) but maladaptation takes over and leads to a reduction in health. On the other hand, if the organism engages its adaptation mechanisms, it increases its health and decreases aspects of Ah but also incurs negative effects such as molecular damage. Thus, aging is perhaps the consequence of the fact that the organism cannot solve the adaptation-maladaptation dilemma permanently, leading to the adaptation-maladaptation theory of aging. An interesting challenge in longevity therapeutics is to solve this conundrum and uncouple beneficial adaptation from harmful adaptation. Another explanation for a reduction in Ab and also for the increase in Ah could be a loss of adaptive precision, meaning that, when challenged, the organism, perhaps as a result of molecular damage, fails to adapt adequately. The organism fails to reach its adaptation goal state with regard to Ab (e.g., it expresses a level of synaptic plasticity below that required for proper memory formation) but instead “lateralizes” its plasticity mechanisms to drive dysfunctional cellular remodeling (e.g., neuronal hyperexcitability). An aging-dependent dysregulation of stimulation-induced transcription has been reported (Martinez-Jimenez et al., 2017).

Another important open question is whether the expression of certain adaptive mechanisms and biomarkers is causative or homeostatic and compensatory. Some adaptive responses could be activated to ensure homeostasis after damage or prevent damage rather than to drive dysfunction themselves. This highlights the importance of direct experimental manipulation of certain markers in addition to association studies.

## Conclusion and outlook

The adaptation-maladaptation framework of aging posits that a cornerstone of aging is a decrease in the ratio of beneficial adaptation to harmful adaptation (Ab/Ah) at several organizational levels of the organism, from cells to cell networks to the whole body. Decreases in Ab lead to lowered capacities in physiological adaptation functions such as learning and memory, immune system plasticity and muscle anabolism, whereas increases in Ah promote dysfunctional metabolic remodeling, cancer, autoimmunity and pathological cardiovascular remodeling among others. Certain adaptation mechanisms such as adaptive transcription can be involved in protection against aging as well as driving aging-related pathologies. Thus, aging-related decline might be inevitable but not necessarily due to random accumulation of damage over time but because adaptive mechanisms will, one way or the other, lead to progressive dysfunction (e.g., either through “directed” damage as part of adaptation or through maladaptation). Because aging is at least partly an active process, it might be possible to counteract it if we understand how biological goal states can be influenced and how adaptation mechanisms can be directed. To do so, we need to study the underlying molecular signaling dynamics in greater detail beyond mere up- or downregulation and beyond simple association studies. Studying aging from the perspective of the adaptation-maladaptation dilemma with the central phenotype of a reduction in Ab/Ah opens up new experimental and theoretical approaches to study longevity mechanisms.

## Data Availability

The original contributions presented in the study are included in the article/supplementary material, further inquiries can be directed to the corresponding author.
